# A rare malignancy mimicker: echinococcus alveolaris

**DOI:** 10.1590/0037-8682-0523-2023

**Published:** 2024-02-05

**Authors:** Elif Gündoğdu, Nevin Aydın

**Affiliations:** 1 Eskişehir Osmangazi University, Faculty of Medicine, Department of Radiology, Eskişehir, Turkey.

**FIGURE 1:**
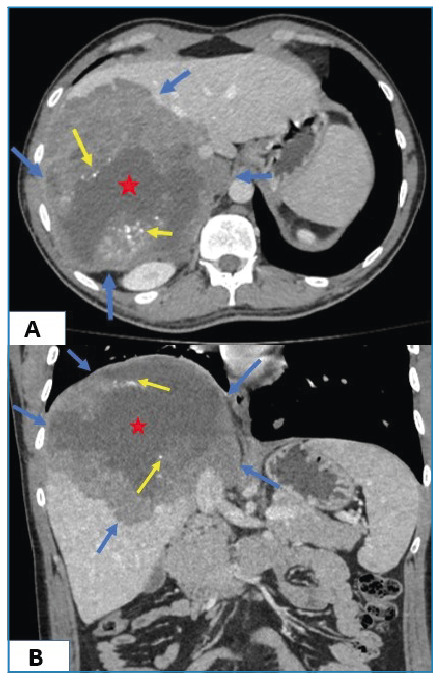
Axial **(A)** and coronal **(B)** plane abdominal CT showing large solitary lesion (blue arrows) with central cystic necrotic areas (stars) and scattered calcified foci (yellow arrows).

**FIGURE 2:**
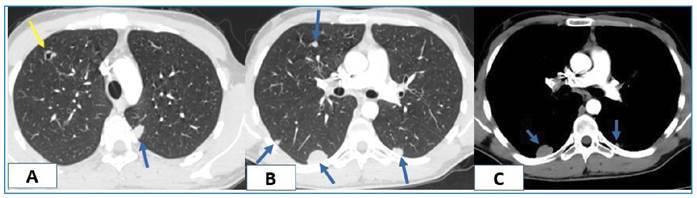
Thoracic CT showing **(A)** cavitary nodule in anterior segment of the upper lobe of the right lung (yellow arrow) and **(B,C)** multiple solid-cystic nodules in both lungs (blue arrows).

A 22-year-old male patient was referred to our radiology department with a suspected metastatic malignancy. His medical history included an intracranial mass found during an examination for headache and visual field blurriness. Complete blood count and biochemical tests revealed no abnormal findings. Elevated erythrocyte sedimentation rate (83 mm/h) and C-reactive protein (125.7 mg/L) levels were detected. Computed tomography (CT) revealed a 14 cm diameter mass in the right lobe of the liver that contained large cystic necrotic areas and scattered calcified foci. Solid soft tissue components were more prominent in the peripheral areas (Figure 1). Thoracic CT showed a cavitary nodule in the anterior segment of the upper lobe of the right lung and multiple solid and cystic nodules in both lungs (Figure 2). Echinococcus alveolaris and metastatic malignancy were considered in the differential diagnosis of the lesions. Echinococcus alveolaris was confirmed histopathologically. Alveolar echinococcosis is a parasitic zoonotic disease caused by echinococcus larvae (metacestodes)^1^. The liver is generally affected, with lung involvement in approximately 13% of cases^2^. Although tumorlike lesions with irregular borders and an infiltrative character with heterogeneous content are typical of liver alveolar echinococcosis, they can be confused with malignancy in disseminated infections^3^. It should be kept in mind during the differential diagnosis, especially in endemic regions.
